# Supporting hemodynamics: what should we target? What treatments should we use?

**DOI:** 10.1186/cc11502

**Published:** 2013-03-12

**Authors:** Luciano Gattinoni, Eleonora Carlesso

**Affiliations:** 1 Dipartimento di Anestesia, Rianimazione (Intensiva e Subintensiva) e Terapia del Dolore, Fondazione IRCCS Ca' Granda - Ospedale Maggiore Policlinico, Via Francesco Sforza 35, 20122 Milan, Italy; 2 Dipartimento di Fisiopatologia Medico-Chirurgica e dei Trapianti, Università degli Studi di Milano, via Francesco Sforza 35, 20122 Milano, Italy

## Abstract

Assessment and monitoring of hemodynamics is a cornerstone in critically ill patients as hemodynamic alteration may become life-threatening in a few minutes. Defining normal values in critically ill patients is not easy, because 'normality' is usually referred to healthy subjects at rest. Defining 'adequate' hemodynamics is easier, which embeds whatever pressure and flow set is sufficient to maintain the aerobic metabolism. We will refer to the unifying hypothesis proposed by Schrier several years ago. Accordingly, the alteration of three independent variables - heart (contractility and rate), vascular tone and intravascular volume - may lead to underfilling of the arterial tree, associated with reduced (as during myocardial infarction or hemorrhage) or expanded (sepsis or cirrhosis) plasma volume. The underfilling is sensed by the arterial baroreceptors, which activate primarily the sympathetic nervous system and renin-angiotensin-aldosterone system, as well as vasopressin, to restore the arterial filling by increasing the vascular tone and retaining sodium and water. Under 'normal' conditions, therefore, the homeostatic system is not activated and water/sodium excretion, heart rate and oxygen extraction are in the range found in normal subjects. When arterial underfilling occurs, the mechanisms are activated (sodium and water retention) - associated with low central venous oxygen saturation (ScvO_2_) if underfilling is caused by low flow/hypovolemia, or with normal/high ScvO_2 _if associated with high flow/hypervolemia. Although the correction of hemodynamics should be towards the correction of the independent determinants, the usual therapy performed is volume infusion. An accepted target is ScvO_2 _>70%, although this ignores the arterial underfilling associated with volume expansion/high flow. For large-volume resuscitation the worst solution is normal saline solution (chloride load, strong ion difference = 0, acidosis). To avoid changes in acid-base equilibrium the strong ion difference of the infused solution should be equal to the baseline bicarbonate concentration.

## Introduction

Hemodynamic assessment is one of the cornerstones of critical care medicine, as hemodynamic alterations may become life-threatening in minutes. Assuring normal hemodynamic values is therefore mandatory to allow one to buy time for patient healing. The problem arises when we have to define normal hemodynamics in critically ill patients. If we are able to define normal hemodynamics, the target values for therapy will follow. In this brief opinion paper we will limit ourselves to the macrohemodynamics and we will discuss the determinants of hemodynamic impairment, the limits of normal, impaired and failing hemodynamics, and the volume therapy to be applied.

## Determinants of hemodynamic impairment

**Figure 1:**
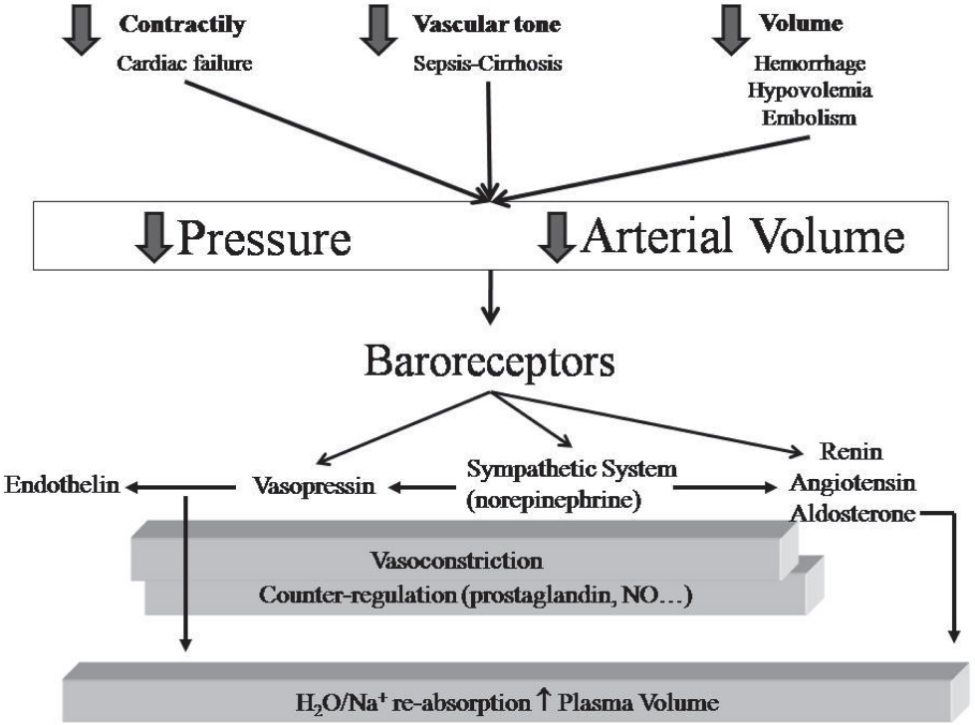
**Independent variables and the sequence of events leading to water and sodium retention**.

We believe the best approach to this issue is the one proposed by Schrier [1], who - based on a series of studies [2,3] - finally introduced a unifying hypothesis to explain the hemodynamic impairment associated with different diseases or syndromes. His approach, slightly modified, is presented in Figure 1, which summarizes the hemodynamic determinants, the neuro-endocrinal signaling and the body response. In this framework we may first consider which variables are independent. These variables belong to three categories: the heart (contractility and heart rate), the vascular tone and, finally, the intravascular volume. In a given disease or syndrome one or more of these variables may be affected. As an example, acute myocardial infarction is paradigmatic of the problems relative to heart contractility and/or rate. A primary alteration of the vascular tone is typical of cirrhosis and septic syndrome. A decreased intravascular volume is typical of hemorrhage. In critically ill patients more than one variable may be altered at the same time, as in sepsis where the impairment of heart contractility and the decrease of intravascular volume due to capillary leakage may be associated with the decrease of the primary artery vessel tone.

We believe that physicians approaching the hemodynamic status of a given patient should first consider which independent determinants are more probably altered. Of interest to note, however, is that, among the primary determinants, the heart rate is the only one always assessed in clinical practice. The contractility measured by echocardiography is occasionally assessed while the vascular tone and the intravascular volume are not measured. One must note that the variables usually evaluated to assess hemodynamics and volemia, such as pressures and flows, are dependent variables that, when altered, may recognize different causes. The identification of or, at least, the estimate of which of the independent hemodynamic determinants is altered makes the therapy a logical consequence. Unfortunately, independent of the altered variable, the first intervention is usually volume replacement. A typical example is represented by hypotension following the induction of anesthesia. In this case the primary cause of hemodynamic impairment is the pharmacologically-induced decrease of the vessel tone but the correction is usually performed by volume infusion.

The baroreceptors, located in the carotid and aortic arch [4], sense the underfilling of the arterial tree (which in a normal situation contains 15% of the intravascular blood volume). This underfilling may be caused either by a decreased intrathoracic volume or cardiac output, as typically occurs during hemorrhage or heart failure, or by arterial vasodilation, as may occur in cirrhosis or sepsis. Interestingly, one must bear in mind that the concept of arterial tree underfilling, which, in some way, recalls the concept of effective circulating blood volume [5,6], may co-exist either with hypovolemia or with hypervolemia, as most of the expanded blood volume may be confined in the venous tract, which acts as a capacitive reservoir. Whatever the cause of the arterial tree underfilling, the body response is similar and primarily consists of activation, via baroreceptors, of the renin-angiotensin-aldosterone system and the nonosmotic release of vasopressin [7]. As shown in Figure 1 other factors may be activated, and a counter-regulation may occur. It is important, however, to realize that the primary body response is directed towards the integrity of the arterial circulation, by maximizing, through the kidneys, the reabsorption of salt and water, while increasing the arterial pressure. There are several elements in favor of this unifying hypothesis, as recently reviewed [7]. Here it is sufficient to say that different diseases or syndromes in which underfilling may occur, with or without plasma expansion, present increased renin, angiotensin and aldosterone levels as well as an increase of vasopressin (anti-diuretic hormone) despite a frequently associated hypo-osmolarity. In general, we believe that the hemodynamic problem and, possibly, the therapeutic interventions may be better understood if considered in this framework.

## Normal hemodynamic, hemodynamic impairment and hemodynamic failure

The concept of normal hemodynamics is not easy to define in critically ill patients. In general, we believe that hemodynamics is adequate when the oxygen delivery to the tissues is sufficient to maintain an aerobic metabolism. This may occur in critically ill patients at hemodynamic values greater than or lower than the values considered normal in healthy subjects at rest. As an example, in cases of decreased hemoglobin content and/or its oxygen saturation, a frequent finding in the ICU, or if hypermetabolism is present, the cardiac output must be greater than normal to provide adequate oxygen transport. In contrast, when the metabolic requirements are reduced, as may occur in critically ill patients during deep sedation or paralysis, the aerobic metabolism may be satisfied with a hemodynamic set of values lower than those considered normal in healthy subjects. In other words, the normality of hemodynamics should not be judged considering the hemodynamic values *per se*, but instead the body response. When the body senses its hemodynamic set as adequate, the baroreceptors may be activated or not activated. If the easily measured variables such as heart rate, urinary output and sodium concentration in the urine remain in the range found in normal subjects, we may assume that the hemodynamics is normal and, obviously, adequate.

**Figure 2:**
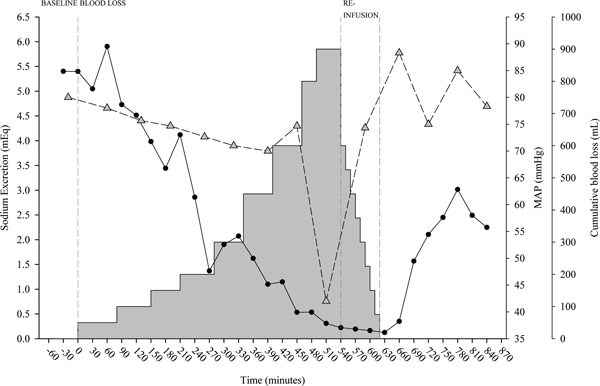
**Sodium excretion and mean arterial pressure during controlled hemorrhage and reinfusion**. The amount of sodium excreted (computed as the product of sodium concentration and urine amount) in 30 minutes (black circles) and the mean arterial pressure (MAP; grey triangles) during controlled hemorrhage and reinfusion (grey area plot) in an experimental swine model. Vertical grey dashed lines indicate the end of baseline/beginning of hemorrhage, end of hemorrhage/beginning of reinfusion and end of reinfusion. As shown, the kidney reacts to the blood volume decrease by retaining sodium and reducing urine volume earlier than significant changes in MAP may be detected. Courtesy of Dr A Protti.

In the presence of abnormal hemodynamic values, either greater or lower than normal, the hemodynamics may be still adequate if it guarantees an aerobic metabolism. This metabolism is obtained by activating all of the homeostatic mechanisms described above. The hemodynamics then becomes inadequate (hemodynamic failure) only when signs of anaerobic metabolism appear, despite the full activation of the mechanisms normally operating to maintain homeostasis. The most recognizable and easy measurable output of such mechanisms are water and sodium retention [1] as judged at least 6 hours after withdrawal of diuretic therapy (too often misused in intensive care). This response, at least in the early phase, should not be confused with kidney failure. On the contrary, the response may be a sign of the maximal response of a normal kidney activated by the sympathetic system and subjected to vasopressin. What is not usually realized is the speed of the change in sodium urine concentration when the system is activated [8]. Figure 2, as an example, presents the electrolyte changes during controlled hemorrhage. As shown, the kidney reacts to the blood volume decrease by retaining sodium earlier than significant changes in mean arterial pressure may be detected.

If the underfilling is caused by decreased blood volume or cardiac output, the water and sodium retention is generally associated with an increased tissue oxygen extraction, as indicated by a decrease in central venous oxygen saturation (ScvO_2_). This is, in our opinion, a reasonable surrogate of the mixed venous saturation [9,10]. We may express ScvO_2 _as a function of its determinants according to the following formula:

$$\text{Scv}{\text{O}}_{\text{2}}=\text{ Sa}{\text{O}}_{\text{2}}\;-\left(\text{V}{\text{O}}_{\text{2}}/\text{Q}\right)\times \text{1}/\text{Hb}$$

As shown, central venous saturation depends on the arterial oxygenation (SaO_2_), on the appropriate match between oxygen consumption/metabolic requirement (VO_2_) and on cardiac output (Q), as well as on the oxygen carrier (Hb). All determinants of oxygen transport, as well as the metabolic rate, may influence the central venous saturation, which is an extremely sensitive, although not specific, indicator of changes in respiratory function (SaO_2_), metabolism (VO_2_), cardiac output (Q) and oxygen carrier (Hb). The physiological meaning of ScvO_2 _may also be expressed as:

$$\text{Scv}{\text{O}}_{\text{2}}=\text{ 1 }-\text{ V}{\text{O}}_{\text{2}}/\text{D}{\text{O}}_{\text{2}}$$

This equation indicates that oxygen venous saturation reflects the residual amount of oxygen in the venous side after consumption (VO_2_) of part of the oxygen delivered (DO_2_). In normal conditions, at rest, the amount of oxygen extracted from the oxygen delivered is about 25% (VO_2_/DO_2_). The ScvO_2 _is therefore around 75%. The arbitrary recommended threshold of ScvO_2 _used in several studies and in guidelines for sepsis treatment is 70% [11-14]. One must note, however, that ScvO_2 _lower than the threshold is not necessarily associated with anaerobic metabolism.

As an example in healthy subjects during physical exercise, ScvO_2 _may decrease to 40% while maintaining aerobiosis, because in this condition cardiac output remarkably increases. The most frequent reason for a decrease in ScvO_2 _in the ICU is cardiac failure. In the framework of the unifying arterial tree underfilling hypothesis, the association between sodium/water retention and low ScvO_2 _is a strong indicator of underfilling due to low flow and/or hypovolemia, as typically observed during heart failure, hemorrhage and dehydration. In contrast, when the underfilling is due to the arterial vasodilatation associated with volume expansion or elevated cardiac output, as in cirrhosis or in some phases of sepsis, ScvO_2 _may be higher than 70 to 75%. Therefore, it is important to realize that even normal or higher than normal ScvO_2 _may be associated with abnormalities of hemodynamics, as assessed by activation of the renin-angiotensin-aldosterone system and vasopressin release. Considering water/sodium retention and hypo-ScvO_2 _or hyper-ScvO_2 _together may therefore indicate whether the arterial tree underfilling is primarily due to low heart contractility/hypovolemia or vasodilatation, respectively. Obviously this is an oversimplification of the problem, but we believe that this approach may provide a reasonable framework when considering the hemodynamic set in a given patient.

When all of the compensatory mechanisms are overcome, the hemodynamic failure is overt and its marker is the appearance of metabolic acidosis associated with increased plasma lactate concentration. We believe that the best approach to understand the relationship between metabolic acidosis and lactate in tissue hypoxia has been provided by Hochachka and Mommsen [15], who elegantly showed how lactate production and ATP hydrolysis are coupled and must be considered together. Here it is enough to say that the appearance of acidosis indicates the energy failure of a group of cells that, in the absence of correction, may result in cellular death after a few hours.

**Figure 3:**
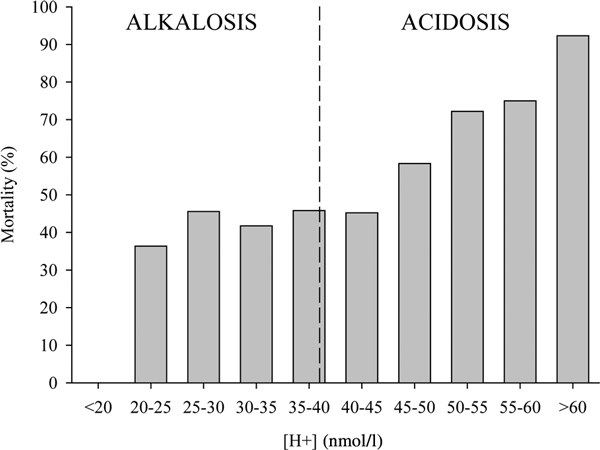
**Relationship between acidosis/alkalosis and mortality in critically ill patients**. Relationship between acidosis/alkalosis (expressed as hydrogen ion concentration) and mortality in a general population of 754 critically ill patients (data recorded at entry to the ICU).

Figure 3 reports the impressive relationship between metabolic acidosis and mortality in a general population of critically ill patients. In contrast, elevated metabolic alkalosis, usually caused by diuretic therapy, is not associated with increased mortality. Beyond pH, several approaches have been proposed to assess the hemodynamic failure [16,17], such as base excess, lactate, decreased strong ion difference (SID), increased anion gap, increased venous to arterial difference in partial pressure of carbon dioxide (PCO_2_) and its ratio to the arterial-venous oxygen content. All these variables, however, are just different facets of the same reality; that is, the perturbation of the acid-base equilibrium due to nonvolatile acid load, originating from suffering cells (lactate) or dead cells (intracellular strong acid content is higher than plasma content). In mammalians, when metabolic acidosis starts due to the hemodynamic failure, the time for correction is limited before irreversible mitochondrial damages and cell death occurs.

## Treatment target

The ultimate goal of the intervention on hemodynamics is to guarantee the maintenance of full aerobic metabolism. The hemodynamic correction may therefore be considered a symptomatic treatment allowing buying time for the cure of the underlying disease. According to Figure 1, the most rational therapy to correct the hemodynamic alterations should be addressed to the correction of the pathogenetic mechanisms; that is, heart contractility/rate, vascular tone or intravascular volume. In clinical practice, independent of the variable primarily altered, the first intervention is usually the volume replacement, according to the following sequence: first, volume; second, cardioactive drugs; and third, blood.

The most popular hemodynamic target, at least in sepsis, is to reach or maintain ScvO_2 _>70% [13,14]. This has been popularized by Rivers and colleagues' study [12], in which the septic patients at entry had baseline ScvO_2 _<50%. Targeting ScvO_2 _of 70%, Rivers and colleagues obtained a significant improvement of the survival rate. In contrast, in a previous study, we could not find any difference in outcome in the same kind of patients with a similar target [11]. The baseline SvO_2 _of these patients however, was 68% - remarkably different from that in Rivers and colleagues' study. We also found that targeting SvO_2 _of 70% was analogous to target a cardiac index of 2.5 l/minute/m^2^. The most plausible explanation for the discrepancy between these studies is the time of intervention: earlier in Rivers and colleagues' study, in the emergency room; and later in our study, after admission to the ICU. However, one should note that all these studies focused on problems associated with a low SvO_2 _state, ignoring the possible hemodynamic derangements occurring in high SvO_2 _states.

## Volume replacement

### Fluid challenge

Volume replacement treatment requires assessment of the patient's intravascular volume status (cardiac preload) and the likelihood of responsiveness (that is, increase the stroke volume) to a fluid challenge test. In fact, data suggest that about 50% of the critically ill patients positively respond to challenge tests [18-20]. Multiple tools have been suggested as indicators for fluid administration, most of them as predictors of response and as targets [21]. Clinical signs, such as thirst, skin turgor, blood pressure, urine output, and so forth, are unreliable indexes of intravascular volume status. Similarly, cardiac filling pressures (central venous pressure (CVP) and pulmonary artery occlusion pressure) that have been traditionally used to guide fluid management are poor predictors [22]. CVP has been used for over 40 years to guide fluid management, as an indicator of intravascular volume (values <8 cmH_2_O indicate hemodynamic impairment), even though this relationship has not been proven. Other techniques, based on echocardiography, such as left ventricular end-diastolic area, or based on thermodilution, such as global end-diastolic volume index, gave unsatisfying results [18].

CVP has been used for decades as an indirect measure of left ventricle preload as it well approximates the right atrial pressure, the major determinant of right ventricle filling [23,24]. Moreover, changes in CVP in response to fluid challenge tests have been used to predict volume responsiveness (target 8 to 12 cmH_2_O) [13]. However, there is increasing evidence that - due to a series of variables, such as venous tone, intrathoracic pressures, ventricular compliances and geometry variations, occurring in critically ill patients - the relationship between CVP and right ventricular end-diastolic volume is poor and that CVP (absolute or changes) does not correlate with volume responsiveness [19]. Similar problems were encountered when referring to the pulmonary artery occlusion pressure [25,26].

During the past decades a number of dynamic tests have been used to dynamically monitor the changes in stroke volume after a maneuver that modifies venous return. These methods have been found more reliable and less invasive than static ones [24].

Heart-lung interaction during mechanical ventilation has been used to evaluate the variations in stroke volume, systolic pressure and pulse pressure. Pulse pressure variation estimated from the arterial waveform and stroke volume variations from pulse contour analysis and pulse oximeter plethysmographic waveform variations have been found to be reliable predictors of a positive response to challenge tests [20]. These hemodynamic effects are due to the cyclic increase/decrease of intrathoracic pressures during mechanical ventilation, affecting right and left ventricular preloads and afterloads. During insufflation, the increased intrathoracic pressures reduce right ventricular stroke volume and increase left ventricular stroke volume. After the blood pulmonary artery transit time (nearly two or three heart beats) even the left ventricular preload decreases with a consequent stroke volume decrease, which is at its minimum value during end expiration. A ventilation-induced change in left ventricle stroke volume of 12 to 13% has been reported highly predictive of volume responsiveness [20]. These methods, however, have some limitations, including the use of tidal volume normalized on ideal body weight >8 ml/kg and the absence of either spontaneous respiratory activity or arrhythmias [27].

Other dynamic tests have been proposed as reliable methods to assess volume replacement responsiveness. These include Doppler echocardiography to assess changes in aortic flow velocity and stroke volume [28,29] and changes in venocaval diameter during positive pressure ventilation estimated by echocardiography [30-33]. The end-expiratory occlusion test consists of the interruption of mechanical ventilation for 15 seconds to suppress the cyclic decrease of cardiac preload during insufflations. The procedure should increase cardiac preload, and an increase of 5% in cardiac output and arterial pulse pressure should predict fluid responsiveness [34]. Finally, passive leg raising has been proposed as an autotransfusion method independent of mechanical ventilation [35]. In conclusion, there is no gold standard clinically available to assess the volume status of the patient. However, the combined use of different methods may provide, in our opinion, an excellent assessment of the hemodynamic status.

### Which fluid?

Three kinds of fluids are available: crystalloids, artificial colloids and albumin. Although a definitive indication of the superiority of one fluid compared with the others is still not available, the data obtained in the last few years have provided, to different extents, some indications. In our opinion, however, discussion of the benefits/risks of the different solutions only applies when large volumes are infused in a relatively short time. Modest infusion, such as 1 to 1.5 l over 24 hours, is likely to be clinically irrelevant. We may roughly divide the effects of the infusion into two main arms: effects due to the volume of the infusion, independent of composition of the solution; and effects due to the quality of the infusion, dependent on the kind of and quantity of solutes present in the fluid replacement.

In critically ill patients, the most general indication for large-volume resuscitation is the refilling of the blood vessels (note that the volume infused is not necessarily proportionally distributed between the arterial and venous trees). Traditionally, we thought that to achieve the same intravascular volume the amount of crystalloids compared with colloids should be in a ratio of 3:1 [13,36]. The most recent large trials comparing colloids and crystalloids, however, indicate that this figure must be corrected - the ratio between crystalloids and colloids, to obtain the same effect, being around 1.5:1 [37-39].

The primary effect of volume is to alter the acid-base status of the blood. This effect becomes clinically relevant when the extracellular fluid dilution is in the order of 10% [40]. We investigated the genesis of acidosis induced by crystalloids in theory [41], *in vitro *[41,42]and *in vivo *[43]. In line with previous results [44-46], we found that dilutional acidosis occurs only when the three determinants of the acid-base status [47,48] - SID, PCO_2 _and total protein content - are unevenly diluted. If these determinants are equally diluted, as during *in vitro *experiments, whatever the composition of the solution used to dilute the plasma (from distilled water to normal saline), the pH does not change if the system is closed because the relative proportions between the pH determinants, equally diluted, are unmodified. If the system *in vitro *is open (by tonometry) to restore the PCO_2 _to that before the dilution, acidosis occurs because the carbon dioxide (volatile acid load) content increases back to the predilution value while the SID and total protein content values remain diluted [41].

Finally, *in vivo*, the SID of the infused solution becomes a determinant to affect the acid-base status [43]. When the SID is lower than the baseline plasma bicarbonate concentration, such as during normal saline infusion, and PCO_2 _is maintained constant, the pH decreases. If the SID of the infused solution is equal to the baseline plasma bicarbonate, acidosis does not develop. On the contrary, if the SID of the infused solution is greater than the baseline plasma bicarbonate concentration, the pH tends to increase [42,43]. The main risks of large crystalloid infusion are therefore edema diffused to the various organs [49] and disturbances of the acid-base equilibrium [40,50,51], depending on the electrolyte composition. With this background, normal saline is the worst approach for large-volume resuscitation; in fact, with the SID of the solution being equal to 0, acidosis is unavoidable. Moreover, the chloride load and the relatively high osmolarity may increase the burden of the kidney with chloride-dependent constriction of the afferent arterioles [52,53].

## Abbreviations

CVP: central venous pressure; PCO_2_: partial pressure of carbon dioxide; SID: strong ion difference; ScvO_2_: central venous oxygen saturation.

## Competing interests

The authors declare that they have no competing interests.

## Declarations

This article has been published as part of *Critical Care *Volume 17 Suppl 1, 2013: Future of Critical Care Medicine. The supplement was proposed by Fresenius Kabi based on presentations from the 'Future of critical care medicine (FCCM) 2012: Today's practice and a look to the future' symposium. Articles were commissioned by the journal, were independently prepared by the authors and have been peer reviewed by the journal. The supplement was edited by John Marini, who declares that he has no competing interests. Publication of the supplement was supported by Fresenius Kabi.
